# Predation Stress Causes Excessive Aggression in Female Mice with Partial Genetic Inactivation of Tryptophan Hydroxylase-2: Evidence for Altered Myelination-Related Processes

**DOI:** 10.3390/cells11061036

**Published:** 2022-03-18

**Authors:** Evgeniy Svirin, Ekaterina Veniaminova, João Pedro Costa-Nunes, Anna Gorlova, Aleksei Umriukhin, Allan V. Kalueff, Andrey Proshin, Daniel C. Anthony, Andrey Nedorubov, Anna Chung Kwan Tse, Susanne Walitza, Lee Wei Lim, Klaus-Peter Lesch, Tatyana Strekalova

**Affiliations:** 1 Department of Psychiatry and Neuropsychology, School for Mental Health and Neuroscience, Maastricht University, 6200 MD Maastricht, The Netherlands; jogikint@gmail.com (E.S.); kplesch@mail.uni-wuerzburg.de (K.-P.L.); 2 Division of Molecular Psychiatry, Center of Mental Health, University of Würzburg, 97080 Würzburg, Germany; 3 Institute of General Pathology and Pathophysiology, Russian Academy of Medical Sciences, 125315 Moscow, Russia; 4 Laboratory of Psychiatric Neurobiology, Institute of Molecular Medicine and Department of Normal Physiology, Sechenov University, 119991 Moscow, Russia; katya.veniaminova@gmail.com (E.V.); jpcosta.nunes@gmail.com (J.P.C.-N.); anna.gorlova204@gmail.com (A.G.); alum1@yandex.ru (A.U.); daniel.anthony@pharm.ox.ac.uk (D.C.A.); 5 Institute of Molecular Medicine, New University of Lisbon, 1649-028 Lisbon, Portugal; 6 Neuroscience Program, Sirius University, 354340 Sochi, Russia; avkalueff@gmail.com; 7 Moscow Institute of Physics and Technology, School of Biological and Medical Physics, 141701 Dolgoprudny, Russia; 8 Institute of Natural Sciences, Ural Federal University, 620002 Yekaterinburg, Russia; 9 P.K. Anokhin Research Institute of Normal Physiology, 125315 Moscow, Russia; proshin_at@mail.ru; 10 Department of Pharmacology, Oxford University, Oxford OX1 3QT, UK; 11 Institute of Translational Medicine and Biotechnology, Sechenov University, 119991 Moscow, Russia; nedorubov.ras@gmail.com; 12 Li Ka Shing Faculty of Medicine, School of Biomedical Sciences, The University of Hong Kong, Pokfulam, Hong Kong SAR, China; annatse@hku.hk; 13 Department for Child and Adolescent Psychiatry and Psychotherapy, University Hospital of Psychiatry Zurich, University of Zurich, 8032 Zurich, Switzerland; susanne.walitza@pukzh.ch

**Keywords:** tryptophan hydroxylase-2 (*Tph2*), female aggression, 5-HT receptors, glycogen synthase kinase-3 β (GSK-3β), myelination, predation stress

## Abstract

The interaction between brain serotonin (5-HT) deficiency and environmental adversity may predispose females to excessive aggression. Specifically, complete inactivation of the gene encoding tryptophan hydroxylase-2 (*Tph2*) results in the absence of neuronal 5-HT synthesis and excessive aggressiveness in both male and female null mutant (*Tph2*^−/−^) mice. In heterozygous male mice (*Tph2*^+/−^), there is a moderate reduction in brain 5-HT levels, and when they are exposed to stress, they exhibit increased aggression. Here, we exposed female *Tph2*^+/−^ mice to a five-day rat predation stress paradigm and assessed their emotionality and social interaction/aggression-like behaviors. *Tph2*^+/−^ females exhibited excessive aggression and increased dominant behavior. Stressed mutants displayed altered gene expression of the 5-HT receptors *Htr1a* and *Htr2a*, glycogen synthase kinase-3 β (*GSK-3β*), and *c-fos* as well as myelination-related transcripts in the prefrontal cortex: myelin basic protein (*Mbp*), proteolipid protein 1 (*Plp1*), myelin-associated glycoprotein (*Mag*), and myelin oligodendrocyte glycoprotein (*Mog*). The expression of the plasticity markers synaptophysin (*Syp*) and cAMP response element binding protein (*Creb*), but not AMPA receptor subunit A2 (*GluA2*), were affected by genotype. Moreover, in a separate experiment, naïve female *Tph2*^+/−^ mice showed signs of enhanced stress resilience in the modified swim test with repeated swimming sessions. Taken together, the combination of a moderate reduction in brain 5-HT with environmental challenges results in behavioral changes in female mice that resemble the aggression-related behavior and resilience seen in stressed male mutants; additionally, the combination is comparable to the phenotype of null mutants lacking neuronal 5-HT. Changes in myelination-associated processes are suspected to underpin the molecular mechanisms leading to aggressive behavior.

## 1. Introduction

Aggression is a behavior that is frequently accompanied by violence, and, as such, results in numerous social problems and adverse health events. The World Health Organization categorizes violent behavior, the incidence of which continues to increase, among the top 20 causes of disability worldwide [1]. Although women are less aggressive than men, female aggression is often expressed in more indirect forms [2]. Recently, an increased incidence of female aggressive behavior in individuals with neuropsychiatric disorders [3] and more frequent crime statistics involving women have been reported [4]. This rise demands a better understanding of the molecular mechanisms that underpin female aggression, but the neurobiology of female aggression is largely unstudied. The use of experimental animal models to investigate the neurobiology of female aggression is limited, as this type of behavior is usually excluded from the normal repertoire of mouse and rat behavioral assessments, and, when it is evaluated, more commonly focuses on male aggression [5,6].

Female aggression can result from a decreased synthesis of neuronal serotonin (5-HT); studies employing complete inactivation of the gene encoding tryptophan hydroxylase-2 (*Tph2*), a key enzyme of 5-HT synthesis in the brain, have revealed that there are higher levels of aggression in female *Tph2*^−/−^ mice [7,8,9,10]. In humans, the *Tph2* gene polymorphism G703T was found to contribute to anger-related traits and the expression of anger in women [11]. Other variants of the *Tph2* gene were also associated with a higher incidence of anxiety disorder in women and with peripartum major depression [12,13].

Accumulating evidence highlights the importance of gene × environment interaction in neuropsychiatric conditions [2,14,15,16,17] and suggests that genetic factors and, for example, a stressful experience, may interact or synergize at a molecular level in the neurobiology of aggression. Mechanistic studies addressing this interaction in the context of female aggression are scarce. Nevertheless, female aggression has been shown to be influenced by environmental adversity, including stress, both in animal experiments [2,6,18] and in clinical studies where verbal and physical aggression was associated with a traumatic stress experience [19].

The relevance of gene × environment interaction in the manifestation of pathological aggression is supported by studies in male mice heterozygous for *Tph2* gene inactivation which exhibits a moderate reduction in brain 5-HT levels of 15–20% [7,8]. *Tph2*^+/−^ mice showed unaltered social behavior at baseline, but, after sub-chronic rat exposure stress, demonstrated markedly increased levels of aggression and dominancy and reduced sociability compared to wild type controls [20,21]. These changes were accompanied by profound alterations in the brain metabolism of 5-HT, dopamine, and norepinephrine. Together, the phenotype of stressed *Tph2*^+/−^ male mice is, therefore, very reminiscent of naïve *Tph2* null mutants.

The effects of environmental challenges and stress on aggression are known to be gender-specific [6]. In rodents, a decrease in aggressive and dominant behaviors has been reported in females subjected to a maternal separation paradigm in C57BL6 mice [22] and in Wistar rats following social isolation stress [23]. Males, by contrast, exhibited increased aggression in these studies. Here, we sought to clarify how gene × environment interactions affect aggressive behavior in female *Tph2*^+/−^ mice and whether aggression in stressed female *Tph2*^+/−^ mice would display similarities to male mutants. Owing to sex differences in the neurobiology of aggression under stressful conditions, we hypothesized that female *Tph2*^+/−^ mice would not demonstrate the abnormally elevated aggressive behavior found in male mutants. We adopted a previously validated five-day rat exposure paradigm, including an element of restraint by virtue of limiting the space available for the free movement of the *Tph2*^+/−^ female mice which has been shown to induce changes in monoamine transmitters, neurogenesis, oxidative stress, as well as aggressive behavior in male *Tph2*^+/−^ mice. This exposure paradigm has been shown to generate similar behavioral changes to those found in another stress protocol variant where animals were placed in larger containers [24]. There is, however, no doubt that immobilizing the mice in the plexiglass tubes will add to the stress experienced, but the approach we adopted reduces the overall number of animals required. The rat exposure procedure applied here has been shown to result in increases in blood levels of CORT in C57BL/6 mice at 6 and 24 h post-stress [24].

In the current study, social interaction/aggression-like behaviors of stressed female mice were scored using measures of home cage social interaction and food competition [25,26,27]. Based on previous findings in *Tph2*^−/−^ males [7,28,29], we studied the gene expression of 5-HT receptors *Htr1a* and *Htr2a*. We also examined the gene expression of glycogen synthase kinase-3β (*GSK-3β*), a marker of distress and degeneration, where changes in expression are known to accompany aberrant serotoninergic processes [30] and regulate aggression and stress responses [31]. Expression of plasticity markers AMPA receptor subunit *GluA2*, synaptophysin (*Syp*), brain-derived neuronal factor (*Bdnf*), its receptor *Trkb*, cAMP response element binding protein (*Creb*), post-synaptic density 95 protein (*PSD95*), and a marker of neuronal activation *c-fos* were also measured [32,33,34]. Gene expression relating to brain myelination was also examined based on our previous findings in stressed male *Tph2*^+/−^ mice [35] where established relationships between myelination and the 5-HT system [36] and stress [37] are recognized. The gene expression of myelin basic protein (*Mbp*), proteolipid protein 1 (*Plp1*), myelin-associated glycoprotein (*Mag*), and myelin oligodendrocyte glycoprotein (*Mog*) was also measured as clinical studies have suggested that elevated aggression is associated with altered myelination in the cortical brain areas [38,39,40,41]. Finally, we sought to determine whether female *Tph2*^+/−^ mice resemble features of *Tph2*^+/−^ males in the broader context of emotional resilience to environmental challenges found in the modified swim test (modFST) and in tests for anxiety-like behavior [20,42] in naïve and stressed female *Tph2*^+/−^ mutants. Potential molecular changes were investigated in the prefrontal cortex, a region of the brain implicated in the mechanisms of both aggression and the response to stress [43,44,45,46,47]. In addition, in the modified swim test, individual predisposition to an enhanced response to adversity learning has been shown to be correlated with molecular changes in the prefrontal cortex which were not observed in the hippocampus [42,48].

## 2. Materials and Methods

### 2.1. The Animals and Housing Conditions

We used 12-week-old *Tph2*^+/−^ female mice, and their wild type littermates, which were bred and genotyped in the facilities at the Institute of Molecular Medicine, New University of Lisbon, Portugal as previously described as controls [8]. Mice of the same genotype were housed in standard cages in groups of five under controlled laboratory conditions (22 ± 1 °C, 55% humidity) and maintained on a reversed 12-h light/dark cycle (lights on at 19:00), with food and water provided ad libitum. All mice were tested during the dark phase of the light/dark cycle. Laboratory housing conditions and experimental procedures were set up and maintained in accordance with Directive 2010/63/EU of 22 September 2010 and had been approved by the Ethics Committee of the New University of Lisbon (No. 0421/000/000/2013). Given that the emotionality and aggression in rodent females are dependent on the estrous cycle, we co-housed the female experimental mice for 4-weeks prior to the start of the experiments with male littermates, which has been previously shown to result in synchronization of the estrous cycle in C57BL6 mice (Veniaminova and Bonapartes, unpublished data). All efforts were undertaken to minimize the potential discomfort of the experimental animals. Experimental protocols conformed to directive 2010/63/EU and were compliant with ARRIVE guidelines (https://arriveguidelines.org accessed on 14 March 2022).

### 2.2. Study Design

**Figure 1:**
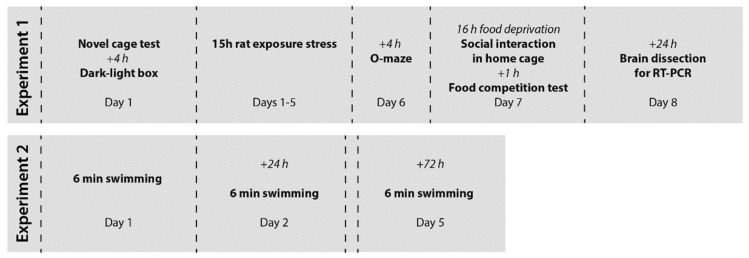
Experiment design. (Experiment 1) Female *Tph2*^+/−^ mice and their wild type littermates were studied for baseline behavior. Thereafter, they were subjected to a five-day rat exposure predation stress model. Mice were studied in a battery of behavioral tests for aggression and anxiety-like behavior before their brains were removed and dissected for qRT-PCR (Experiment 2). A separate cohort of mice was used for the modFST. qRT-PCR—quantitative reverse transcription polymerase chain reaction assay.

Female *Tph2*^+/−^ mice and their wild type littermates (*Tph2*^+/+^ controls) were studied for baseline behavior in novel cage and dark-light box paradigms (Figure 1, Experiment 1). Mice from four cages per genotype were studied: two cages per genotype per stress condition. Thereafter, they were subjected to a five-day rat exposure predation stress model and social behavior was evaluated in their home cages, in food competition tests, and on the elevated O-maze. The sequence of the behavioral tests was designed in a manner to minimize any potential effects of the testing procedure on the experimental animals and the outcome of the subsequent tests [49,50]. In total, mice from four cages per genotype were studied: two cages per genotype per stress condition. Mice were sacrificed 24 h after the last behavioral test and their brains were dissected for qRT-PCR assay. During this study, daily food intake was monitored (see below). A separate cohort of mice was studied in the modFST in which the animals were exposed to three 6-min swim sessions on days 1, 2, and 5. The learning of adverse context is defined by an increase in floating behavior from day 2 to day 5 (Figure 1, Experiment 2) [42]. On average, 7–10 animals per group were used for behavioral and molecular assays, group sizes are indicated in figure legends.

### 2.3. Novel Cage

The vertical exploratory activity of mice was studied in the novel cage test under a red light as previously described [34,50,51]. Briefly, mice were placed into a plastic cage and the number of exploratory rears was counted during a five-minute period under red light.

### 2.4. Dark-Light Box

The dark-light box (Open Science, Moscow, Russia) consisted of two plexiglass compartments, a dark box (15 × 20 × 25 cm) and a light box (30 × 20 × 25 cm), connected by a tunnel. Mice were placed into the dark compartment, from where they could visit the light compartment, illuminated by bright light (300 lx intensity). The total duration of time spent in the light compartment was scored over 5 min [52].

### 2.5. Rat Exposure Stress

Mice were introduced into a transparent glass cylinder (15 cm high × 8 cm diameter) and placed into the rat cage between 18:00 and 9:00 for five consecutive nights as described elsewhere [20,24]. Mice had free access to food and water in their home cages between the stress sessions. The timing of the rat exposure model was designed to minimize the impact of food and water deprivation, as the predation period overlaps with the light (inactive) phase of activity of the mice when food and water consumption is minimal [53,54]. As the analysis of aggressive behavior in *Tph2*^+/−^ male mice that were exposed to a five-day predation stress regimen only exhibited a significant increase of aggressiveness on day 5 [21], we considered the same five-day stress procedure as minimally sufficient for the induction of aggression in the current study.

### 2.6. Home Cage Interaction

In all experimental groups, dominant, aggressive, and other social behaviors in a home cage were assessed during a ten-minute period under low lighting (5 lx) after 16 hours of food deprivation. In this study, daily food intake was measured three days prior to and one day after the behavioral test. The top of a home cage was replaced by a transparent cover and mice were scored for the latency, total duration and number of episodes of crawl-over, following and agonistic (attacking) behaviors, and the number of mice expressing these behaviors [25,26]. The social interaction behavioral parameters recorded and evaluated here have been validated in previous studies on female mice [26].

The crawl-over behavior, considered as a manifestation of hierarchical dominance [55,56,57], was defined as the movement of a mouse over the body of the partner; predominantly headfirst crossing transversely from one side to the other [56,58]. Following behavior, another sign of hierarchical dominance in female mice [59], was defined as the aggressive and rapid chasing of a fleeing counter-partner where the maximum distance between the animals was one body length (adapted from [57]). Agonistic (attacking) behavior was defined by the occurrence of a physical attack of one mouse against another which involved kicking, wrestling, biting, or rolling over the body of the counter-partner (adapted from [60,61]).

### 2.7. Food Competition Test

The food competition test was carried out immediately after the recording of the home cage behavior (see Section 2.6). Pairs of 16 h food-deprived mice from different cages and the same experimental group were placed in a plastic observation cage (21 ×  27 × 14 cm) and allowed to compete for a piece of beef meat (2 g) for 10 min under low lighting (5 lx). The number and duration of attacks were scored [25,26]. The same definitions of social behavior as in the home cage interaction situation were used; these parameters were validated in previous studies on female mice [25].

### 2.8. Elevated O-maze

The apparatus (Open Science, Moscow, Russia) consisted of a circular path (runway width 5.5 cm, diameter 46 cm) that was placed 45 cm above the floor. Two opposing arms were protected by walls (closed area, height 10 cm). The apparatus was placed on a dark surface to maintain control over lighting conditions during testing, which was kept constant at 25 lux. Mice were placed in one of the closed-arm areas of the apparatus. Behavior was assessed using previously validated parameters during a 5-min observation period. The latency to the first exit into the open arms of the maze, the number of exits into the open arms, and time spent in the open arms were all recorded [62].

### 2.9. Modified Forced Swim Test

The modified forced swim test (modFST) was used here as a model that seeks to mimic the neurobiological changes that involve the enhanced learning of adversities and result in helplessness in a particular context [42]. Mice were subjected to two swimming sessions with an interval of 24 h. After the first two swim sessions, a third swim session was carried out on day 5 as previously described [42,63,64]. All sessions were 6-min long and were performed by placing a mouse in a transparent cylinder (⌀ 17 cm) filled with water (23°C, water height 13 cm, the height of cylinder 20 cm). The floating behavior was defined as the absence of any directed movements of the head or body and was scored by an observer unaware of the identity of the animal with Noldus EthoVision XT 8.5 (Noldus Information Technology, Wageningen, The Netherlands) as described elsewhere [65]. The duration of floating behavior was assessed in 2-min intervals; the latency to float was measured. It is of note that in this model, the increase in floating behavior, which is observed on day 5 compared to day 2, is reversible by pre-treatment with antidepressant compounds [48,64,66]. For this reason, the increase in day 5 floating is regarded as a measure of excessive conditional learning and helplessness in an adverse context [63,64]. The increase in floating behavior during the first observation interval from day 2 to day 5 was expressed as a percentage and interpreted as a measure of learning in an adverse context and helplessness [48,63,64].

### 2.10. Brain Dissection and Tissue Collection

Mice were terminally anesthetized with an intraperitoneal injection of sodium pentobarbitone (Merck, Darmstadt, Germany); the left ventricle was perfused with 10 mL of ice-cold saline [51]. The brains were removed and the prefrontal cortex was isolated and stored at −80 °C as described elsewhere [21,67].

### 2.11. Quantitative Real-Time PCR (qRT-PCR)

RNA extraction and cDNA synthesis were performed as described elsewhere [68]. Total mRNA was isolated from each sample with TRI Reagent (Invitrogen, Carlsbad, CA, USA). During first-strand cDNA synthesis, 1 μg total RNA was converted into cDNA using random primers and Superscript III transcriptase (Invitrogen, Carlsbad, CA, USA). qRT-PCR was performed using the SYBR Green master mix (Bio-Rad Laboratories, Philadelphia, PA, USA). qRT-PCR was performed in a 10 μL reaction volume containing a SYBR Green master mix (5 μL), RNase-free water (3 μL), specific forward and reverse primers used at the concentration 20 pmol/μL (1 μL), and cDNA (1 μL). The initial denaturation step for qRT-PCR was at 95 °C for 5 min followed by 40 cycles of denaturation at 95 °C for 30 s and annealing at 60 °C for 30 s. The sequences of primers used are listed in Appendix A Table A1; all primers were purchased from Life Technologies (Carlsbad, CA, USA). All samples were run in triplicate. Relative gene expression was calculated using the ΔΔCt method and normalized to the expression of glyceraldehyde 3-phosphate dehydrogenase (*GAPDH*) as the housekeeping gene and the expression of the control sample as described elsewhere [34,69]. For technical reasons, i.e., owing to the limited amount of cDNA that was available for the PCR assays, the numbers of samples used in the RT-PCR assays are variable, but the sample allocation was performed before any analysis was performed.

### 2.12. Statistical Analysis

Data analysis was performed using GraphPad Prism software version 8.3 (San Diego CA, USA). Normally distributed data were analyzed using an unpaired Student’s *t*-test or a two-way ANOVA test followed by the Tukey’s correction for the pairwise comparisons of the group means of behavioral and molecular data. Specifically, the Tukey’s test was used for the post-hoc analysis of gene expression results, as each RT PCR assay in this study was carried out separately for each transcript and because the confidence intervals obtained for the values of the mRNA concentrations and the fold changes of all investigated transcripts do not include zero values. Nonparametric data were analyzed by Kruskal-Wallis test and Dunn’s post-hoc test. Fisher’s exact test was performed for analysis of contingency tables. Statistical significance was set at *p* < 0.05. Data are shown as mean ± SEM.

## 3. Results

### 3.1. The Predation Stress Procedure Induces Aggressive and Dominant Behavior in Tph2^+/−^ Females

**Figure 2:**
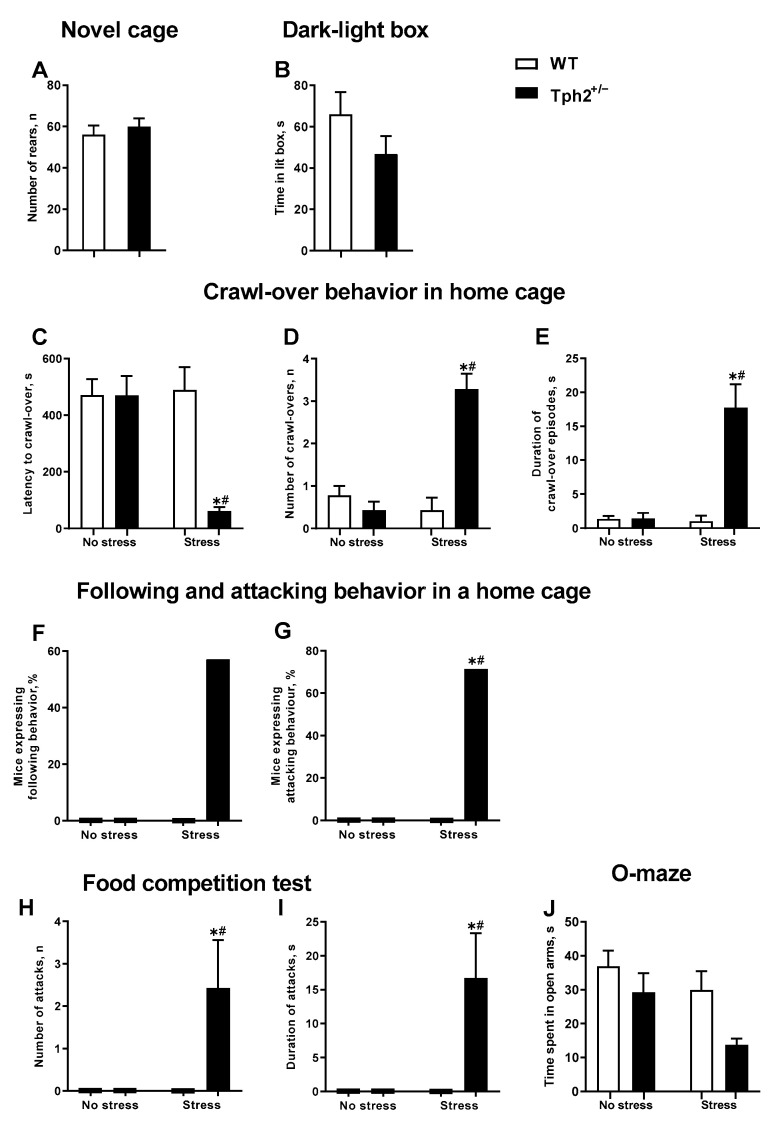
Behavioral features of naïve and stressed *Tph2*^+/−^ female mice. (**A**) No alteration in the exploratory behavior of naïve *Tph2*^+/−^ mice was found in the novel cage test and (**B**) in the time spent in the lit box (controls: *n* = 14, mutants: *n* = 10). (**C**) Significantly lower latency to crawl-over, significantly elevated number of crawl-overs (**D**), and duration of crawl-over behavior (**E**) in the social interaction in the home cage were present in the stressed *Tph2*^+/−^ group. (**F**) There was no significant group difference in the percentage of the animals exhibiting the following behavior in social interactions in the home cage. (**G**) In social interactions in the home cage, agonistic behavior was displayed by a significantly higher percentage of animals in the stressed *Tph2*^+/−^ group, in comparison with non-stressed *Tph2*^+/−^ mice or stressed wild type animals. (**H**) In the food competition test, a significantly greater number and (**I**) duration of attacks were observed in the stressed *Tph2*^+/−^ group. (**J**) No significant group differences in the time spent in the open arms were found in the O-maze (**C**–**J**) (no stress: *n* = 9; stress, *n* = 7). WT—*Tph2*^+/+^, * *p* < 0.05 vs. same-genotype non-stressed group, # *p* < 0.05 vs. stress-matched WT group.

In the novel cage test, the number of exploratory rears did not differ significantly between the *Tph2*^+/+^ and *Tph2*^+/−^ mice (t = 0.6140, df = 22, *p* = 0.55, unpaired *t*-test. Figure 2A). The time spent in the lit box of the dark-light box test was also not significantly different between these groups (t = 1.378, df = 18, *p* = 0.19, unpaired *t*-test. Figure 2B).

The latency to crawl-over, number of crawl-overs, and total duration of this behavior, as a measure of home cage dominance, were significantly different between the groups as studied in the home cage (H = 15.14, *p* < 0.01, H = 17.73, *p* < 0.01 and H = 17.39, *p* < 0.01, respectively; Kruskal-Wallis test. Figure 2C–E). The latency to crawl-over in the stressed *Tph2*^+/−^ group was significantly shorter in comparison to both non-stressed *Tph2*^+/−^ and stressed *Tph2*^+/+^ (wild type) animals (both *p* < 0.01, Dunn’s test). The number of episodes and the duration of crawl-over behavior were significantly higher in the stressed mutant mice in comparison to non-stressed *Tph2*^+/−^ animals and stressed controls (all *p* < 0.01). While there was no significant group difference in the number of animals displaying the following behavior (all *p* = 0.07, Fisher’s exact test. Figure 2F), in comparison to both non-stressed *Tph2*^+/−^ and stressed *Tph2*^+/+^ mice, the number of animals displaying agonistic (attacking) behavior was significantly higher in the stressed *Tph2*^+/−^ group (both *p* = 0.02, Figure 2G). None of the non-stressed mice exhibited following or attacking behaviors, regardless of the genotype (Figure 2F,G).

**Figure A1:**
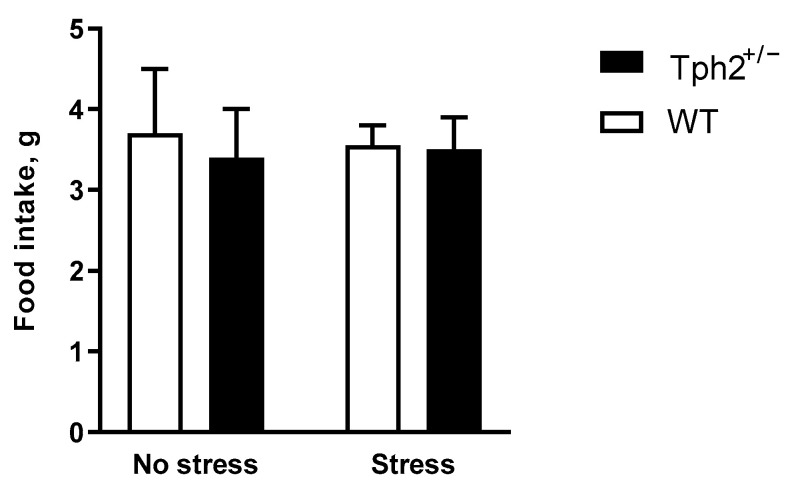
Daily food intake of *Tph2*^+/−^ mice. No significant group difference in average daily food intake during the observation period was observed. WT—wild type.

In the food competition test, significant differences were found between the groups in both the number and the duration of attacks (H = 14.57, *p* < 0.01, and H = 14.57, *p* < 0.01, respectively. Figure 2H,I). Post-hoc analysis revealed that, in comparison to both non-stressed *Tph2*^+/−^ group and stressed *Tph2*^+/+^ mice, the number and duration of attacks were significantly elevated in the stressed *Tph2*^+/−^ group (both *p* = 0.01, Dunn’s test). In a similar manner to the home cage assay, none of the non-stressed mice exhibited following or attacking behaviors in the food competition test, regardless of the genotype (Figure 2H,I). In the O-maze, Kruskal-Wallis testing showed a significant group difference in the time spent in the open arms (H = 14.19, *p* < 0.01. Figure 2J). The only significant difference was found between the non-stressed wild type mice and stressed mutants (*p* < 0.01); post-hoc analysis did not show significant differences between genotype-matched or stress-matched groups. The Kruskal-Wallis test did not demonstrate any significant group differences in the food intake (H = 0.17, *p* = 0.99, Kruskal-Wallis test. Figure A1).

### 3.2. Altered Gene Expression of Selected Molecular Markers in the Prefrontal Cortex of Stressed Tph2^+/−^ Mice

**Figure 3:**
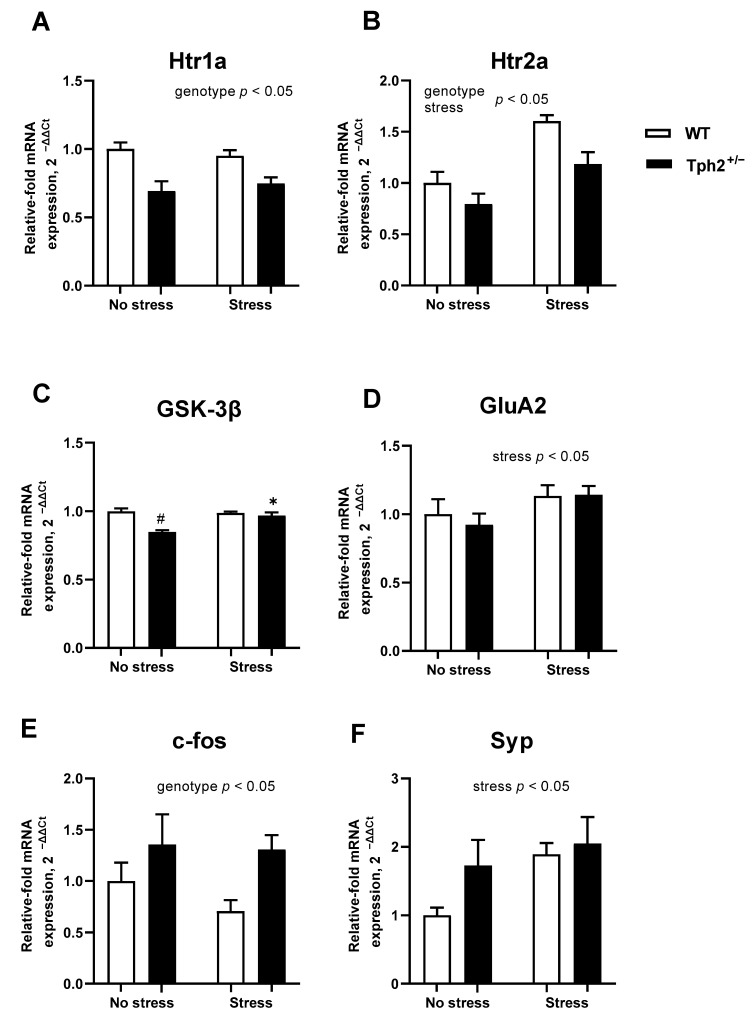
Expression of 5-HT receptors, *GSK-3β*, *GluA2*, *c-fos* and *Syp* in the brain of stressed *Tph2*^+/−^ mice. (**A**) Compared to control groups, *Htr1a* expression was significantly lowered in *Tph2*^+/−^ animals. WT no stress (NS) *n* = 4, WT stress (S) *n* = 9, *Tph2*^+/−^ NS *n* = 6, *Tph2*^+/−^ S *n* = 6. (**B**) In comparison to non-stressed animals, in stressed groups, *Htr2a* expression was significantly higher. Irrespectively of stress, *Htr2a* expression was higher in wild type groups. WT NS *n* = 4, WT S *n* = 8, *Tph2*^+/−^ NS *n* = 6, *Tph2*^+/−^ S *n* = 6. (**C**) Significantly higher *GSK-3β* expression in both the stressed *Tph2*^+/−^ group and non-stressed *Tph2*^+/+^ mice was observed in comparison to non-stressed *Tph2*^+/−^ animals. WT NS *n* = 4, WT S *n* = 6, *Tph2*^+/−^ NS *n* = 6, *Tph2*^+/−^ S *n* = 4. (**D**) A significant main effect of stress was observed for the *GluA2* subunit, where expression was elevated independent of the genotype in stressed groups. WT NS *n* = 5, WT S *n* = 9, *Tph2*^+/−^ NS *n* = 6, *Tph2*^+/−^ S *n* = 6. (**E**) Expression of the *c-fos* was higher in *Tph2*^+/−^ mice than in wild-type mice, irrespective of stress. WT NS *n* = 5, WT S *n* = 9, *Tph2*^+/−^ NS *n* = 6, *Tph2*^+/−^ S *n* = 6. (**F**) In stressed animals, expression of *Syp* was higher than in non-stressed animals, irrespectively of the genotype. WT NS *n* = 6, WT S *n* = 9, *Tph2*^+/−^ NS *n* = 5, *Tph2*^+/−^ S *n* = 6. WT—wild type, * *p* < 0.05 vs. same-genotype non-stressed group, # *p* < 0.05 vs. stress-matched WT group.

Two-way ANOVA revealed a significant main effect of genotype (F1,21 = 21.40, *p* < 0.01) and no significant stress × genotype interaction (F1,21 = 0.93, *p* = 0.35) in *Htr1a* expression. Independent of stress, a significant decrease in the expression of the *Htr1a* was found in *Tph2*^+/−^ animals (Figure 3A). No significant stress × genotype interaction was shown in the expression of *Htr2a* (F1,20 = 1.240, *p* = 0.28, two-way ANOVA. Figure 3B), though both main effects of stress and genotype significant altered expression (F1,20 = 26.58, *p* < 0.01, and F1,20 = 10.59, *p* < 0.01, respectively, two-way ANOVA). *Htr2a* expression was significantly higher in the stressed animals, which was independent of genotype, but lower in the mutant groups, independent of stress. These data suggest differential regulation of expression of 5-HT receptor subtypes by stress and partial *Tph2* inactivation.

For *GSK-3β* expression, a significant stress × genotype interaction was observed (F1,16 = 16.47, *p* < 0.01, two-way ANOVA. Figure 3C). In comparison to non-stressed *Tph2*^+/−^ animals, post-hoc analysis revealed significantly higher *GSK-3β* expression in both the stressed *Tph2*^+/−^ group and the non-stressed *Tph2*^+/+^ mice (both *p* < 0.01, Tukey’s test). *GluA2* expression was not significantly affected by stress × genotype interaction (F1,22 = 0.248, *p* = 0.62. Figure 3D) and only a significant main effect of stress was observed (F1,22 = 4.331, *p* = 0.05). Specifically, stress elevated *GluA2* expression compared to non-stressed animals irrespective of their genotype.

No stress × genotype interaction was found for either *c-fos* or *Syp* expression (F1,22 = 0.437, *p* = 0.52, and F1,22 = 1.149, *p* = 0.30, respectively, two-way ANOVA), though the main effects of genotype or stress on gene expression were observed. The expression of *c-fos* was significantly higher in the *Tph2*^+/−^ mice in comparison with control animals, independent of stress (F1,22 = 6.63, *p* = 0.02, two-way ANOVA. Figure 3E). The expression of *Syp* was significantly higher in stressed animals than in controls (F1,22 = 5.24, *p* = 0.03. Figure 3F), independent of genotype.

**Figure A2:**
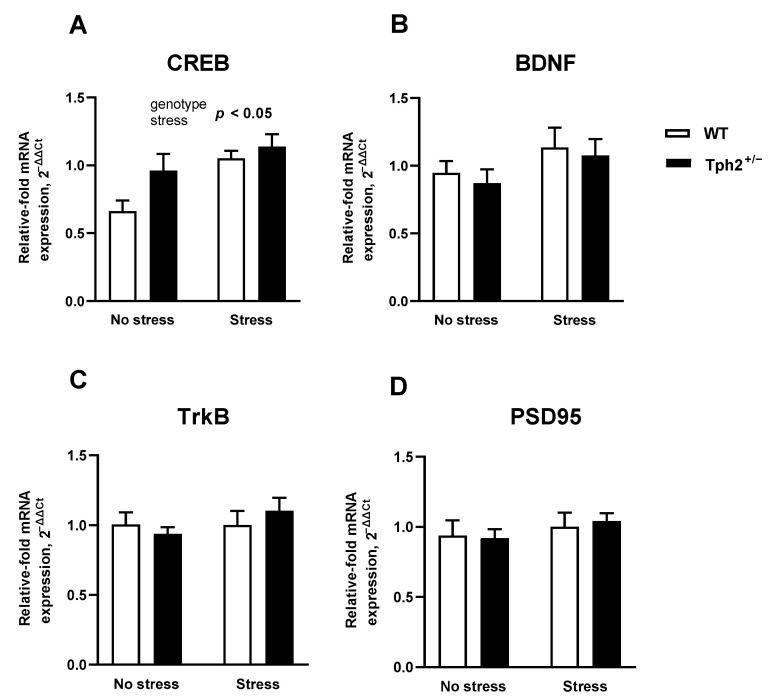
Expression of *Creb*, *Bdnf*, *Trkb* and PSD95 in the prefrontal cortex of *Tph2*^+/−^ mice. (**A**) *Creb* expression was significantly higher in the stressed animals, independent of genotype (WT NS *n* = 6, WT S *n* = 9, *Tph2*^+/−^ NS *n* = 6, *Tph2*^+/−^ S *n* = 6). (**B**) No significant differences were found for *Bdnf* expression (WT NS *n* = 6, WT S *n* = 9, *Tph2*^+/−^ NS *n* = 6, *Tph2*^+/−^ S *n* = 7), (**C**) *Trkb* expression (WT NS *n* = 6, WT S *n* = 9, *Tph2*^+/−^ NS *n* = 6, *Tph2*^+/−^ S *n* = 7), or for (**D**) PSD95 expression (WT NS *n* = 6, WT S *n* = 9, *Tph2*^+/−^ NS *n* = 6, *Tph2*^+/−^ S *n* = 7). WT, wild type.

Two-way ANOVA revealed significant main effects for genotype and stress (F1,23 = 4.87, *p* = 0.04 and F1,23 = 10.38, *p* < 0.01, respectively, two-way ANOVA), but there was no stress × genotype interaction (F1,23 = 1.46, *p* = 0.24) for *Creb* expression. This measure was significantly higher in the stressed animals and was independent of the genotype; in the mutant groups, it was independent of the stress (Figure A2A). These data suggest the differential regulation of expression of *Creb* by stress and partial *Tph2* inactivation.

There was no significant stress × genotype interaction and no significant main effects of genotype or stress on *Bdnf* expression (F1,24 = 0.0047, *p* = 0.95; F1,24 = 0.28, *p* = 0.60 and F1,24 = 2.29, *p* = 0.14, respectively; Figure A2B), *Trkb* expression (F1,24 = 0.868, *p* = 0.36; F1,24 = 0.039, *p* = 0.85 and F1,24 = 0.76, *p* = 0.39, respectively; Figure A2C), or for the expression of PSD95 (F1,24 = 0.106, *p* = 0.95; F1,24 = 0.018, *p* = 0.89 and F1,24 = 1.025, *p* = 0.32, respectively; Figure A2D).

**Figure 4:**
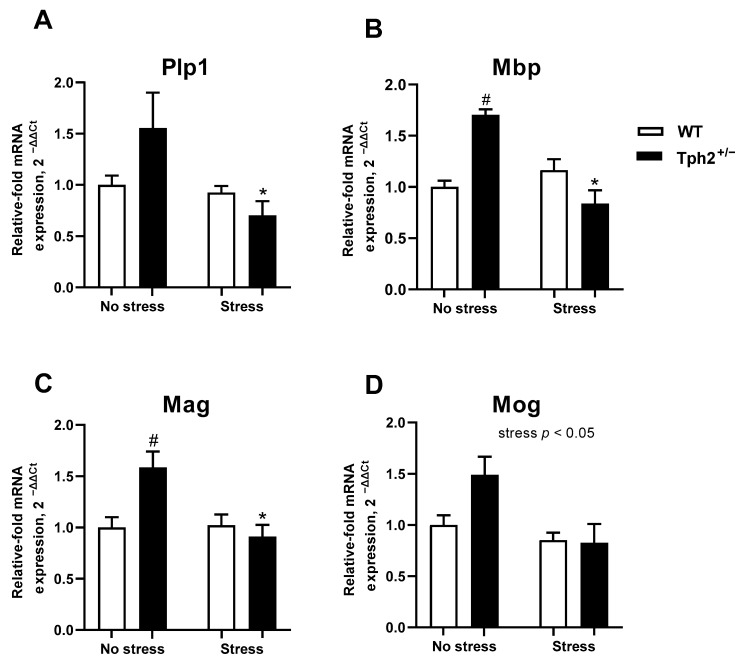
Elevated expression of myelination-related genes in the prefrontal cortex of non-stressed *Tph2*^+/−^ mice. (**A**) Significantly lower expression of *Plp1* was observed in stressed *Tph2*^+/−^ mice in comparison to the non-stressed *Tph2*^+/−^ group. WT no stress (NS) *n* = 5, WT stress (S) *n* = 9, *Tph2*^+/−^ NS *n* = 5, *Tph2*^+/−^ S *n* = 4. (**B**,**C**) Compared to non-stressed *Tph2*^+/−^ group, expression of *Mbp* and *Mag* was significantly lower in both stressed *Tph2*^+/−^ and non-stressed *Tph2*^+/+^ mice. *Mbp*: WT NS *n* = 4, WT S *n* = 9, *Tph2*^+/−^ NS *n* = 3, *Tph2*^+/−^ S *n* = 4. *Mag*: WT NS *n* = 5, WT S *n* = 9, *Tph2*^+/−^ NS *n* = 4, *Tph2*^+/−^ S *n* = 4. (**D**) In comparison to non-stressed mice, stressed animals had a significantly lower expression level of *Mog*, irrespective of the genotype. WT NS *n* = 5, WT S *n* = 9, *Tph2*^+/−^ NS *n* = 4, *Tph2*^+/−^ S *n* = 5. WT—wild type, * *p* < 0.05 vs. same-genotype non-stressed group, # *p* < 0.05 vs. stress-matched WT group.

A stress × genotype interaction exists for *Plp1* expression (F1,19 = 4.949, *p* = 0.04, two-way ANOVA). Post-hoc analysis revealed significantly lower expression of *Plp1* in stressed *Tph2*^+/−^ mice in comparison to non-stressed *Tph2*^+/−^ mice (*p* = 0.02, Tukey’s test, Figure 4A). No significant differences were observed between *Tph2*^+/+^ stressed and naïve mice (*p* = 0.07). For *Mbp* and *Mag* expression, ANOVA revealed significant stress × genotype interaction (F1,16 = 16.68, *p* < 0.01 and F1,18 = 7.610, *p* = 0.01 respectively, Figure 4B,C). Compared to the non-stressed *Tph2*^+/−^ group, the expression of *Mbp* and *Mag* was significantly lower in both stressed *Tph2*^+/−^ (*p* = 0.01 and *p* = 0.02, respectively, Tukey’s test) and non-stressed *Tph2*^+/+^ mice (*p* < 0.01 and *p* = 0.03, respectively). ANOVA revealed no significant interaction for *Mog* expression (F1,19 = 4.098, *p* = 0.06, two-way ANOVA. Figure 4D), though a significant main effect of stress was observed (F1,19 = 10.08, *p* < 0.01). In comparison to non-stressed mice, stressed animals had a significantly lower expression level of *Mog*, irrespective of their genotype.

### 3.3. Naïve Female Tph2^+/−^ Mice Show Signs of Decreased Learning of Adverse Memories and Helplessness as a Manifestation of Stress Resilience

**Figure A3:**
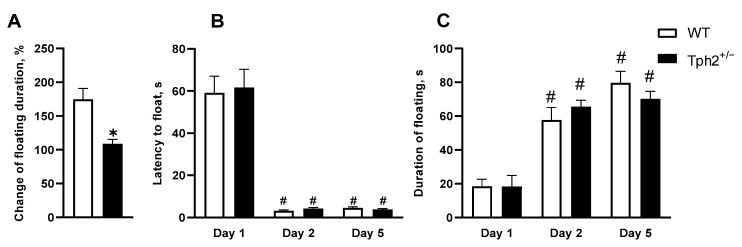
Floating behavior in the modified swim test. (**A**) A smaller increase in floating duration from day 2 to day 5 was observed in the *Tph2*^+/−^ mice compared to WT. (**B**) A significant decrease in latency to float on days 2 and 5 compared to day 1 was observed and was independent of genotype. (**C**) There was a significant increase in the duration of floating on days 2 and 5 compared to day 1, independent of the genotype. WT—wild type, * *p* < 0.01 vs. wild type, # *p* < 0.01 vs. same genotype on day 1. WT no stress *n* = 13, WT stress *n* = 13, *Tph2*^+/−^ NS *n* = 11, *Tph2*^+/−^ S *n* = 12.

In the modFST, in comparison to wild type mice, *Tph2*^+/−^ mice demonstrated a significantly smaller increase in floating duration in the first two minutes of the test session between days 2 and 5 (U = 15, *p* < 0.01, Mann-Whitney test; Figure A3A). In the latency to float and the duration of floating, there was no significant interaction between day and genotype, though a main effect of the test day was found (F1,14 = 91.79 and F1,12 = 89.22, respectively, both *p* < 0.01, repeated measures two-way ANOVA; Figure A3B,C). No significant group differences in the latency and duration of floating were found on either day of the test.

## 4. Discussion

Our study has revealed that aggressive and dominant behaviors are induced in female *Tph2*^+/−^ mice subjected to predation stress, resembling a behavioral profile reported for stressed male *Tph2*^+/−^ mutants and mice with complete inactivation of *Tph2*. Wild type stressed controls did not show any of these changes. We also found a decrease in gene expression of *Plp1*, *Mbp*, and *Mag* in the prefrontal cortex of stressed mutants, which may reflect aberrant myelination processes which likely to contribute to stress-induced aggression and dominance behavior. Baseline expression of *GSK-3β* was lower in the non-stressed *Tph2*^+/−^ mice than in the wild type animals. Unlike wild type mice, mutants showed relatively increased *GSK-3β* expression under stress conditions. The lowered basal expression of *GSK-3β* in female *Tph2*^+/−^ mutants may also explain a diminished increase in behavioral despair during repeated swimming in the modFST, a sign of stress resilience.

The increased aggression and dominance in stressed mutants were accompanied by genotype effects on the prefrontal cortex expression of *Htr1a* and *Htr2a*. Both receptors are known to modulate aggressive behavior [70,71,72]. The expression of *Htr1a* and *Htr2a* were decreased in *Tph2*^+/−^ females regardless of stress, which is also a feature of *Tph2*^−/−^ mutants; it might be explained by a higher sensitivity of this receptor, at a protein level, to diminished levels of central 5-HT [73]. However, in the *Tph2*^+/−^ males subjected to predation stress there was no effect on *Htr1a* or *Htr2a* expression. For *Htr1a*, the sex-dependent behavioral effects, which have been reported after the pharmacological targeting of 5-HT_1A_ receptor in rodents [74], suggest that there is likely to be a differential role for this receptor in abnormal aggression in males and females.

The predation stress paradigm used in this work was previously shown to increase 5-HT turnover in the amygdala of male *Tph2*^+/−^ mice [21]. Furthermore, significantly elevated 5-HT turnover in the prefrontal cortex of stressed male *Tph2*^+/−^ mice correlated with measures of aggressiveness (Bazhenova and Lesch, *unpublished results*). Surprisingly, stressed *Tph2*^+/−^ males exhibited unaltered 5-HT levels in the prefrontal cortex, while wild type mice showed significant increases in 5-HT levels under these conditions. These abnormalities might arise from the compromised 5-HT metabolism in the prefrontal cortex of stressed mutants that results in disrupted cortical top-down control of limbic structures regulating aggression, including the amygdala, and thus, these changes could underpin the social abnormalities observed in the stressed female *Tph2*^+/−^ mice.

As compromised serotonin metabolism in the *Tph2*^+/−^ mutants can independently result in the altered regulation of appetite, satiety, and metabolic processes, in which changes in monoamine levels and changes in the expression of their receptors can play a major role [75], the excessive aggression in stressed mutants in our study might be food deprivation-state-dependent. Preliminary studies on *Tph2*^+/−^ stressed mice, housed under normal conditions, did not reveal any changes in social behavior in the food competition test (Strekalova and Costa-Nunes, unpublished results). In the present study, we used a food deprivation challenge, a well-established inducer of aggression in male mice [76,77], and hierarchical dominance behaviors in female mice [59]. Further studies are warranted to address the issue as to how the changes in serotonin receptor expression and the effects of food deprivation and aggression in stressed *Tph2*^+/−^ mice are related.

Genetic deficits in 5-HT function are well-established to result in developmental abnormalities of brain connectivity [36,78,79,80]. Compromised frontostriatal white matter integrity and connectivity are believed to underlie increased impulsivity and aggression [41,81,82]. Here, for the first time, we report the increased expression of genes encoding myelination-related proteins in the prefrontal cortex of naïve *Tph2*^+/−^ female mice and its significant decrease following predation stress. Previous work has shown that there is decreased expression of *Mbp* and *Mag* in naïve *Tph2*^+/−^ males [35]. Thus, the present findings in naïve *Tph2*^+/−^ females may mirror compensatory effects such as the elevated expression of myelin genes that is neutralized by stress, leading to impaired connectivity and maladaptive aggression in these animals. The stress-induced decrease of myelination-related marker expression was previously reported in other rodent models of stress, such as chronic unpredictable stress, social defeat and social isolation, immobilization stress, and early-life stress [83,84].

Moreover, others have previously demonstrated a relationship between myelination in the prefrontal cortex and aggression and emotional dysregulation. Reduced thickness of the myelin sheath in the prefrontal cortex was reported to correlate with increased aggression caused by juvenile isolation [85]. Group housing was shown to ameliorate both aggressive behaviors and the myelination deficit in another study of social isolation in mice [37,86]. In rats, the overexpression of the myelin transcription factor 1 (MyT1) promotes differentiation of oligodendrocytes, which is also regulated by Plp1 and Mbp [87], and ameliorates anxiety-like and compulsive behaviors [88]. Aberrant myelination is believed to underlie impaired brain connectivity and be associated with impulsive and aggressive behaviors, contributing to neurodevelopmental disorders such as attention deficit hyperactivity disorder (ADHD), autism spectrum disorders (ASD), and schizophrenia [89,90]. We may speculate that the changes observed in the expression of myelin associated transcripts in stressed *Tph2*^+/−^ mice may reflect developmental abnormalities of white matter and brain connectivity and, though unlikely to be the sole cause of the excessive aggression observed in these mice, may contribute to behavior. This view is further supported by clinical evidence. For example, in women with ADHD and borderline personality disorder, there are correlations between anger-hostility measures and impairments of inferior frontal white matter connectivity [38]. Reduced white matter volume in the frontostriatal tracts, particularly in medial prefrontal regions, was associated with increased impulsivity in healthy subjects maturing from their adolescence to adulthood [41]. Aggression scores correlated with fronto-accumbal white matter integrity and cortical thickness of the orbitofrontal cortex in children with ADHD [39]. In patients recovering from mild traumatic brain injury, reduced fiber integrity in the white matter also correlates with higher measures of aggression [40].

Other molecular processes may potentially contribute to the abnormal social behavior of stressed *Tph2*^+/−^ mice. Genotype differences in the expression of brain *c-fos* argue for a role of this factor in the aggressive behavior of stressed female *Tph2*^+/−^mice. In males, by comparison, *c-fos* expression was increased in the amygdala and prefrontal cortex of stressed mice of both genotypes [21]. Over-expression of *c-fos* in the hippocampus of *Tph2*^−/−^ mice is accompanied by increased freezing in the fear conditioning paradigm; a trend towards both molecular and behavioral changes was reported in the *Tph2*^+/−^ mutants [8,91]. It can be speculated that the increased expression of this immediate early gene, as found in the stressed *Tph2*^+/−^ groups 24 h after the last manipulation, might be related to increased conditioning after the handling procedure. While chronic stress has been reported to suppress the expression of *Syp*, a marker of neuronal plasticity [92,93], here, *Creb* expression was elevated in female *Tph2*^+/−^ mice regardless of stress exposure. This may indicate compensatory plasticity processes related to the up-regulation of myelination in naïve mutants and may further contribute to their stress resilience as shown in the modFST. Indeed, increased CREB activity was previously associated with elevated aggression in female mice [94,95]. While the expression of *Creb* was shown to be related to levels of BDNF and its receptor [96,97,98], mRNA levels of *Bdnf* and *Trkb* were unaltered in this study, as well as gene expression of PSD95, which have been correlated with increased aggression in female rodents in other studies [99]. These results suggest that more complex regulatory interactions underpin emotional control than those described by these plasticity markers alone in the prefrontal cortex.

Upregulated myelination markers may also relate to the decreased baseline expression of *GSK-3β*, a key indicator of helplessness behavior in naïve mutants [42]. Previous studies point to a reciprocal relationship between GSK-3β and myelination-related factors, e.g., Mbp [100,101], that is in keeping with our findings of increased gene expression of the latter molecules found in naïve mutants. It is of note that decreased basal expression of *GSK-3β* in the female *Tph2*^+/−^ mutants may also contribute to the smaller increase in behavioral despair during repeated swimming in the modFST. Previous studies have revealed an important role of increased brain GSK-3β activities in subgroups of mice that display susceptible, but not resilient, responses in this model [42]. In effect, mice that display a prolongation of the floating behavior from day 2 to day 5 above mean values for the group exhibit increased mRNA concentration for *GSK-3β*, decreased levels of phosphorylated GSK-3β at 9-serine, and a reduced ratio of phosphorylated GSK-3β to overall GSK-3β content, i.e., increased GSK-3β activity, in the prefrontal cortex [42,48]. These behavioral and molecular changes were reduced by pre-treatment with low doses of imipramine or anti-oxidant compounds [48,63,64,68]. Therefore, the lowered baseline expression of *GSK-3β* in the pre-frontal cortex of *Tph2*^+/−^ mutants might explain the smaller increase in behavioral despair observed during repeated swimming in the modified swim test. Notably, a functional interaction was previously reported between decreased *Tph2* enzymatic activity and GSK-3β in male mice with knock-in of the human R439H mutation [102].

Concerning potential mechanisms for a lower stress/despair response of female *Tph2*^+/−^ mutants in the modified swim test, we hypothesize that this might also be due to the suppression of the expression of 5-HT1A and 5-HT2A receptors in the brain, whose roles in stress response, major depressive disorder, and consolidation of aversive memories are well established [70,103,104,105]. Furthermore, it can be speculated that in a similar fashion to male *Tph2*^+/−^ mutants that exhibit ‘stress resilience’ in the modFST [20], female *Tph2*^+/−^ mice exhibit altered dopamine metabolism; turnover of dopamine in major mesocorticolimbic regions can govern individual susceptibility to stress [106,107] and was particularly marked in female mice [108].

In the present study, stress-induced increases of expression of *GSK-3β* and *GluA2* were not affected by the mutation. Similar results were found in the brain of stressed *Tph2*^+/−^ males for *GSK-3β*, but *GluA2* was upregulated selectively in the male mutants [21]. This challenges the view that these transcripts play a pivotal role in the aggression elicited in stressed *Tph2*^+/−^ females [24,33] and further suggests that sex differences result in the differential regulation of aggression ein *Tph2*^+/−^ mice. For GSK-3β, given that the level of the phosphorylated form of this kinase is the principal determinant of its activity, activity has been shown to correlate with *GSK-3β* gene expression changes [109]. However, further assessment of the level of GSK-3β phosphorylation might be useful to confirm this association and its role in the behavioral abnormalities of the *Tph2*^+/−^ females reported here.

## 5. Conclusions

Taken together: our findings show that an interaction between partial genetic inactivation of neuronal *Tph2* expression and environmental adversity results in aggressive and dominant behaviors in female *Tph2*^+/−^ mice. Naïve female *Tph2*^+/−^ mice show decreased learning of adverse memories and helplessness, a sign of stress resilience. These behaviors are reminiscent of changes in *Tph2*^+/−^ males and null mutants of both sexes lacking *Tph2*. For the first time, we report the altered expression of myelination markers in naïve and stressed female *Tph2*^+/−^ mice. These data encourage speculation regarding impaired brain connectivity in these mice, which likely contributes to the increased aggression and dominance observed in the stressed *Tph2*^+/−^ mice. Further studies are required to shed light on the detailed mechanisms of the relationships between serotonin deficiency, stress, and myelination in the context of gene × environment interaction and female aggression.

## Data Availability

Data supporting reported results can be obtained on a request. Alternatively, they can be obtained via the links to publicly archived datasets analyzed and generated during the study (https://www.sechenov.ru/univers/structure/nauchno-tekhnologicheskiy-park-biomeditsiny/instituty/institut-molekulyarnoy-meditsiny/laboratorii/psikhneiro, accessed on 14 March 2022).

## Appendix A

### Appendix A.1. Supplementary Methods

**Table A1:** Primer sequences for mRNA expression analysis.

Gene	Primer	Sequence
*Htr1a*	Forward	5′-GACAGGCGGCAACGATACT-3′
	Reverse	5′-CCAAGGAGCCGATGAGATAGTT-3′
*Htr2a*	Forward	5′-TAATGCAATTAGGTGACGACTCG-3′
	Reverse	5′-GCAGGAGAGGTTGGTTCTGTTT-3′
*GSK*-*3**β*	Forward	5′-GCACTCTTCAACTTTACCACTCA-3′
	Reverse	5′-CGAGCATGTGGAGGGATAAG-3′
*GluA2*	Forward	5′-GCGTGGAAATAGAAAGGGCC-3′
	Reverse	5′-ACTCCAGTACCCAATCTTCCG-3′
*c-fos*	Forward	5′-CGGGTTTCAACGCCGACTA-3′
	Reverse	5′-TTGGCACTAGAGACGGACAGA-3′
*Syp*	Forward	5′-TGTGTTTGCCTTCCTCTACTC-3′
	Reverse	5′-TCAGTGGCCATCTTCACATC-3′
*Plp1*	Forward	5′-CCAGAATGTATGGTGTTCTCCC-3′
	Reverse	5′-GGCCCATGAGTTTAAGGACG-3′
*Mbp*	Forward	5′-TCACAGCGATCCAAGTACCTG-3′
	Reverse	5′-CCCCTGTCACCGCTAAAGAA-3′
*Mag*	Forward	5′-GGTACATGGCGTCTGGTATTTC-3′
	Reverse	5′-ACTTGTGTGCGGGACTTGAAG-3′
*Mog*	Forward	5′-TCATGCAGCTATGCAGGACAA-3′
	Reverse	5′-TTTCGGTAGAGGTGAACCACT-3′
*Creb*	Forward	5′-CAGGGGTCGCAAGGATTGAAG-3′
	Reverse	5′-ATCGCCTGAGGCAGTGTACT-3′
*Bdnf*	Forward	5′-TGGCTGACACTTTTGAGCAC-3′
	Reverse	5′-AAGTGTACAAGTCCGCGTCC-3′
*Trkb*	Forward	5′-CCTCCACGGATGTTGCTGAC-3′
	Reverse	5′-GCAACATCACCAGCAGGCA-3′
*PSD-95*	Forward	5′-GACGCCAGCGACGAAGAG-3′
	Reverse	5′-CTCGACCCGCCGTTTG-3′
*GAPDH*	Forward	5′-ATGACCACAGTCCATGCCATC -3′
	Reverse	5′-GAGCTTCCCGTTCAGCTCTG-3′

### Appendix A.2. Supplementary Results

#### Daily Food Intake of *Tph2*^+/−^ Mice

The Kruskal-Wallis test did not reveal significant differences in the average daily food intake measured during the observation period (H = 0.17, *p* = 0.99, Kruskal-Wallis test. Figure A1).

### Appendix A.3. Expression of Neurotrophic Factors in the Prefrontal Cortex of Stressed Tph2^+/−^

The two-way ANOVA and post-hoc comparisons revealed group differences in the expression of neurotrophic molecules in the brains of the experimental groups (see ms main text; Figure A2).

### Appendix A.4. Tph2^+/−^ Mice Display Reduced Potentiation of Floating in the modFST Paradigm

The change in floating duration in the first two minutes of the test session between days 2 and 5 in *Tph2*^+/−^ animals was significantly smaller than in wild type mice (see ms text, Figure A3A). Concerning the latency to float and the duration of floating, only the main effect of the test day was found (see ms text, Figure A3B,C). Post-hoc analysis revealed a significant decrease in latency to float and a significant increase in the duration of floating on days 2 and 5 compared to day 1, irrespective of the genotype (both *p* < 0.01, Šídák’s multiple comparisons test).
